# Supplementary material for: Motifs of the C-terminal domain of MCM9 direct localization to sites of mitomycin-C damage for RAD51 recruitment
Source: J Biol Chem. 2021 Feb 2;296:100355. doi: 10.1016/j.jbc.2021.100355 (PMC7949153; doi:10.1016/j.jbc.2021.100355)
Supplement: Supplemental Figures S1–S7 and Table S1 [file mmc1.pdf]

Supporting Information

Motifs of the C-terminal domain of MCM9 direct localization to sites of mitomycin-C damage for RAD51 recruitment

David R. McKinzey, Shivasankari Gomathinayagam, Wezley C. Griffin, Kathleen N. Klinzing, Elizabeth P. Jeffries, Aleksandar Rajkovic, and Michael A. Trakselis

Experimental procedures:

Live/Dead Cell Determination: Viability of HEK293T, MCM8^{KO}, and MCM9^{KO} cells was determined using propidium iodide as previously described (1). HEK293T cells and respective knockout cells were treated with 3 µM MMC for 24 hours. Cells were washed with PBS twice and treated with PBS trypsin solution for 10 minutes. Cells were collected by centrifugation at 300 xg for 5 minutes and washed three times with PBS. Staining of the cells was carried out by resuspending and incubating in 0.5 mg/mL propidium iodide in PBS for 15 minutes in the dark. PI staining signal intensity was obtained using a FACS Verse instrument (BD Biosciences, San Jose, CA). Data was collected using the following parameters: 488 nm laser for excitation, 630/22 nm filter for emission, with at least 10,000 events recorded. All data was evaluated using FLOWJO v10 (BD Biosciences).

References

1. Crowley, L. C., Scott, A. P., Marfell, B. J., Boughaba, J. A., Chojnowski, G., and Waterhouse, N. J. (2016) Measuring Cell Death by Propidium Iodide Uptake and Flow Cytometry. *Cold Spring Harb Protoc* **2016**

**Supplementary Table S1: DNA Sequences**	
**DNA**	**Sequence (5’-3’)**
MCM9For*XhoI*	ATTACTCGAGCATGAATAGCGATAAG
MCM9648Rev*XmaI*	ATTACCCGGGCTATGACTTTTTTCTCATCTC
MCM9605For*XhoI*	CACCCTCGAGGGGAGGTGCACTGCTAGGAGGT
MCM9Rev*XmaI*Stop	ATAGATCCCGGGCTATGACTTTTTTCTCATCTCTTCATCC
MCM9NLS1QCF	CAACCACAGCTCCAATGCGTGTCAGTGATGATGCATCTTTTCAGCTCCGTGGGTCCACCG
MCM9NLS1QCR	GCAAGGGATTTAGGCCTGTCATCTTTGCCTGAGTCCAGCCTTCTGTCTGGGCTATGTACAG
MCM9NLS2QCF	GGGCAGACAATGTGGAAAGTAACAAGGATGATAGACTAGCACTAGATTCTGAAGCAGCAG
MCM9NLS2QCR	CTGCTGCTTCAGAATCTAGTGCTAGTCTATCATCCTTGTTACTTTCCACATTGTCTGCCC
MCM9NLS3QCF	GTTCATAGTCCTAAAATTTCCCAGGATGATGATACTAGTAGAGACGCAGCCTTGCCGGTGAAG
MCM9NLS3QCR	CTTCACCGGCAAGGCTGCGTCTCTACTAGTATCATCCTGGGAAATTTTAGGACTATGAAC
MCM9NLS4QCF	CGGTGGACCCACGGAGCTGAAAAGATGCATCATCACTGACACGCATTGGAGCTGTGGTTG
MCM9NLS4QCR	CAACCACAGCTCCAATGCGTGTCAGTGATGATGCATCTTTTCAGCTCCGTGGGTCCACCG
MCM9BRCvF687A/R688AQCF	GGTCCAGGGGAAGAATCAAACGCCGCGACGTCATCACAGCAGGAAATCAA
MCM9BRCvF687A/R688AQCR	TTGATTTCCTGCTGTGATGACGTCGCGGCGTTTGATTCTTCCCCTGGACC
MCM9BRCvF687EQCF	TGAGAAATGGTCCAGGGGAAGAATCAAACGAGCGTACGTCATCACAGCAGGAAATCAACTATAGCAC
MCM9BRCvF687EQCR	GTGCTATAGTTGATTTCCTGCTGTGATGACGTACGCTCGTTTGATTCTTCCCCTGGACCATTTCTCA
MCM9BRCvL720AQCF	CTAGATCCCCCACCGCATGCGGAGCCTAATAGATCA
MCM9BRCvL720AQCR	TGATCTATTAGGCTCCGCATGCGGTGGGGGATCTAG
MCM9BRCvCOF	CTGCGTAATGGTCCTGGTGAGGAAAGCAACGCAGCAACAAGCAGCCAGCAGGAGATTAACTATAGCACC
MCM9BRCvCOR	GGTGCTATAGTTAATCTCCTGCTGGCTGCTTGTTGCTGCGTTGCTTTCCTCACCAGGACCATTACGCAG
MCM9643COFor*BamHI*TEV	TTATTAGGATCCGAGAATCTATACTTTCAAGGTAGCCTGCTGAGTGAGGAGCTGCGTCGT
MCM9680COFor*BamHI*TEV	TTATTAGGATCCGAGAATCTATACTTTCAAGGTGGTCCTGGTGAGGAAAGCAACTTCCG
MCM9900CORev*XhoI*	TTATTACTCGAGGGCTAAGCTCTTAGGACGTTTGCGCTTC
MCM9620FNdeI	ATTACATATGCCTGAAAACCCTGGAGAGCAG
MCM91143RSalI	ATTAGTCGACCTATGACTTTTTTCTCATCTC
RAD51For*MfeI*	ATTACAATTGATGGAAAGCTTTGGCCCACAACCC
RAD51Rev*XhoI*	ATTACTCGAGTCTGTCTTTGGCATCTCCCACTCCATC
siRAD51	GAGUUGACAAACUACUUC

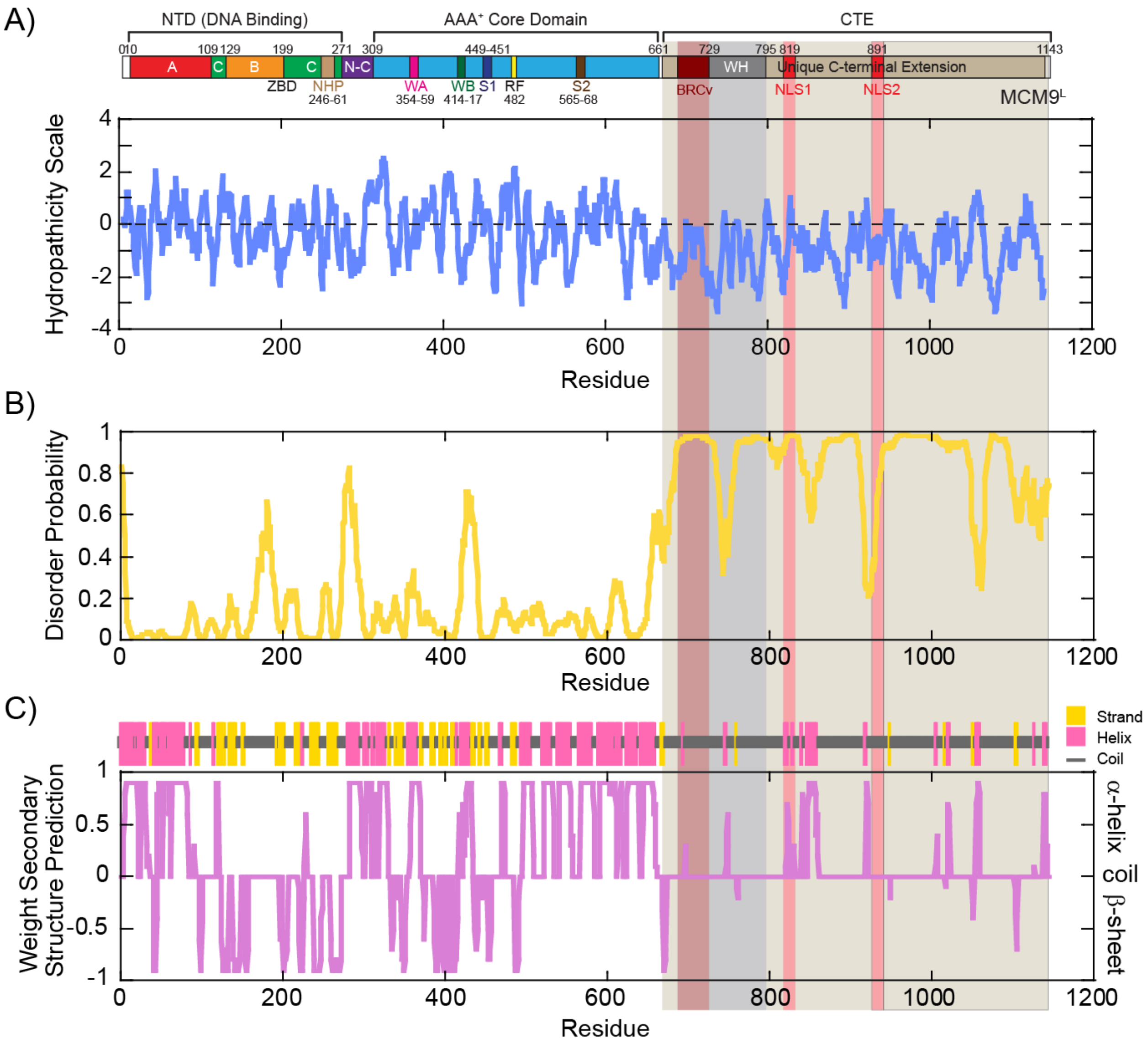

Supplementary Figure S1. Structural prediction of the MCM9 sequence show an unstructured CTE. The MCM9 primary protein sequence was analyzed using A) ProtScale (https://web.expasy.org/protscale/) for hydrophathicity, B) DISOPRED (http://bioinf.cs.ucl.ac.uk/disopred/) for predicted disorder, and C) PSIPRED (http://bioinf.cs.ucl.ac.uk/psipred/) for secondary structure prediction. The C-terminal half of the protein including the CTE (shaded beige) is expected to be mostly hydrophobic, disordered, and unstructured.

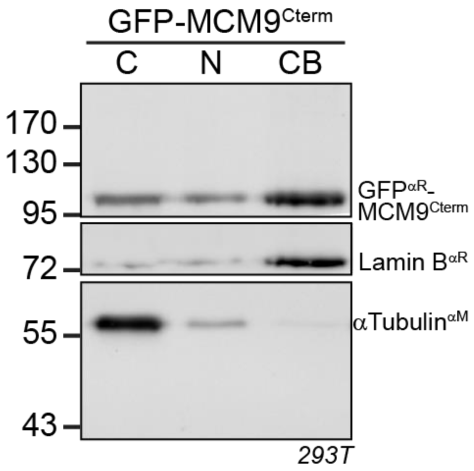

Supplemental Figure S2: MCM9^{Cterm} binds chromatin. Western blot of cytoplasmic (C), nuclear (N), and chromatin bound (CB) extractions of GFP transfected MCM9^{Cterm} in 293T cells after treatment with MMC. Controls are included for nuclear (Lamin B) and cytoplasmic (tubulin) fractions.

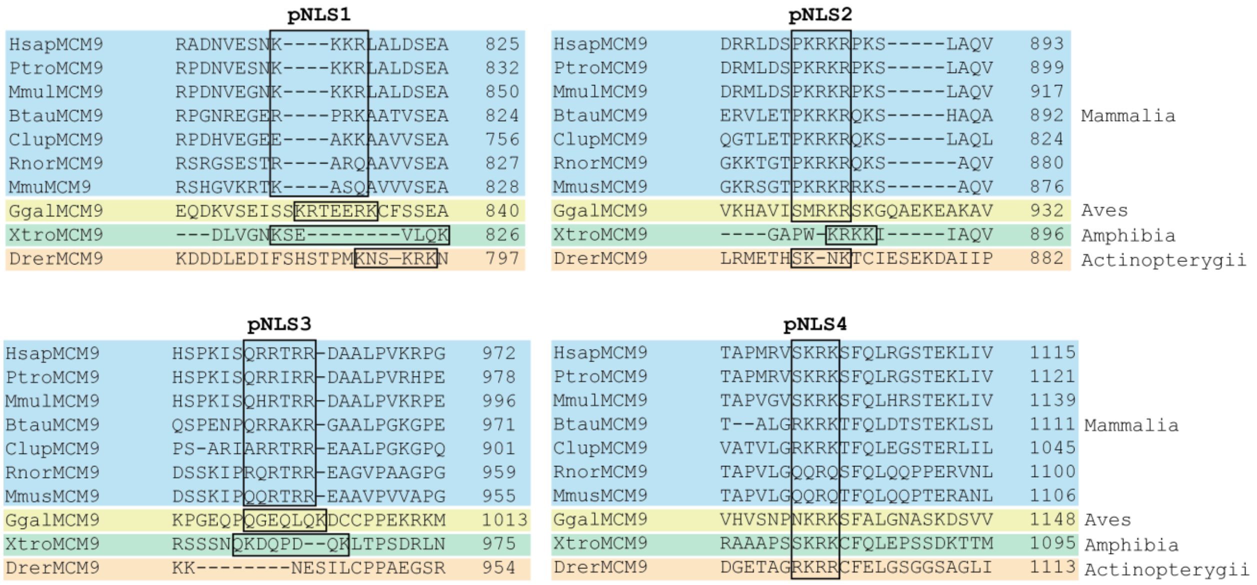

Supplementary Figure S3. Alignments of the NLS sequences across *Mammalia*, *Aves*, *Amphibia*, and *Actinoptergii* species. Black boxes indicate potential homology.

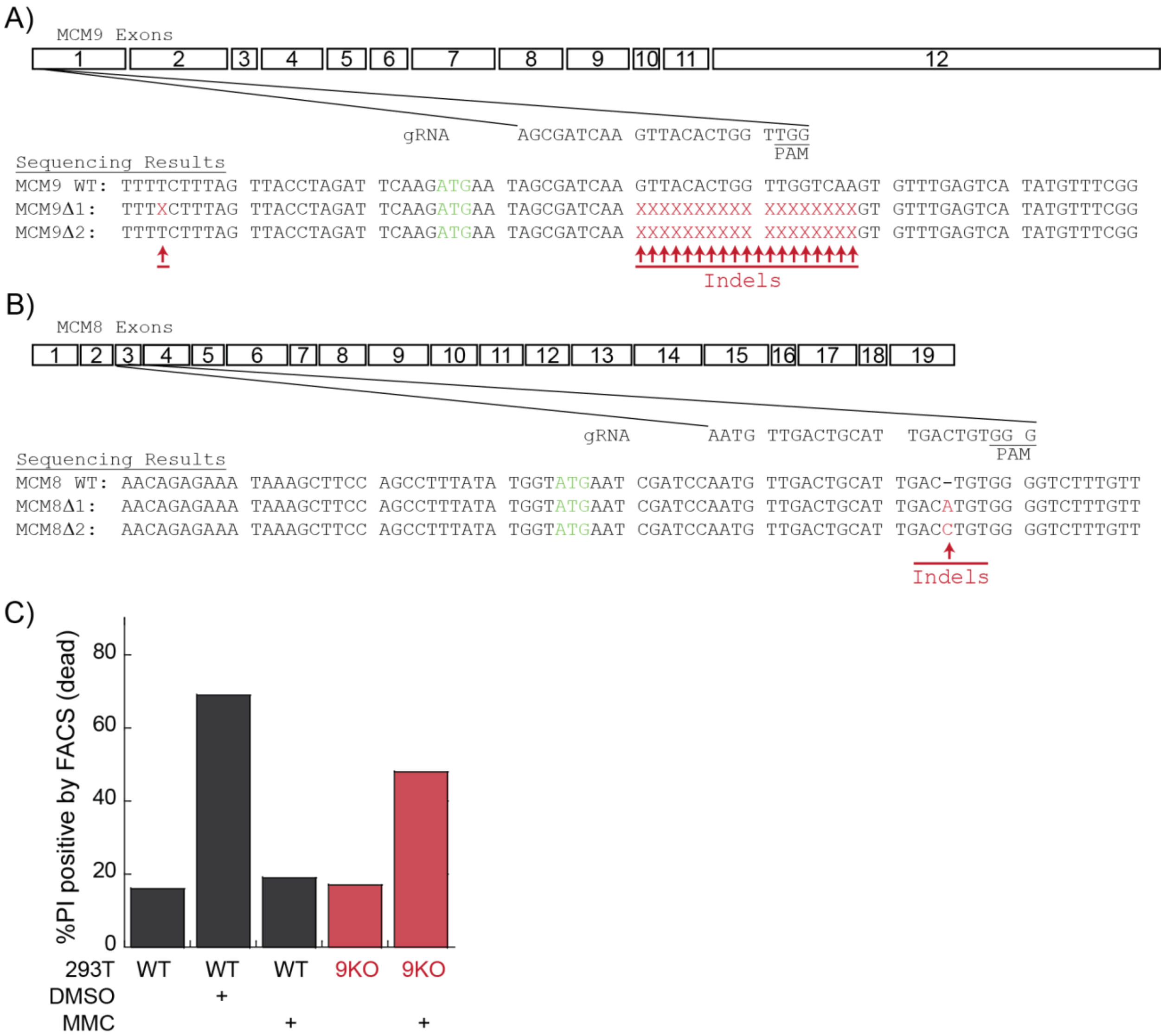

Supplemental Figure S4: CRISPR-Cas9 knockout strategy and validation of MCM8 and MCM9 in HEK293T cells. CRISPR/Cas9 targeting of A) exon 1 of MCM9 or B) Exon 3 of MCM8 and DNA sequencing results for the selected clones highlighting indels. C) Quantification of dead cells for WT 293T or MCM9^{KO} (9KO) after treatment with 3 μM MMC. DMSO was used as a positive control for cell death in WT 293T.

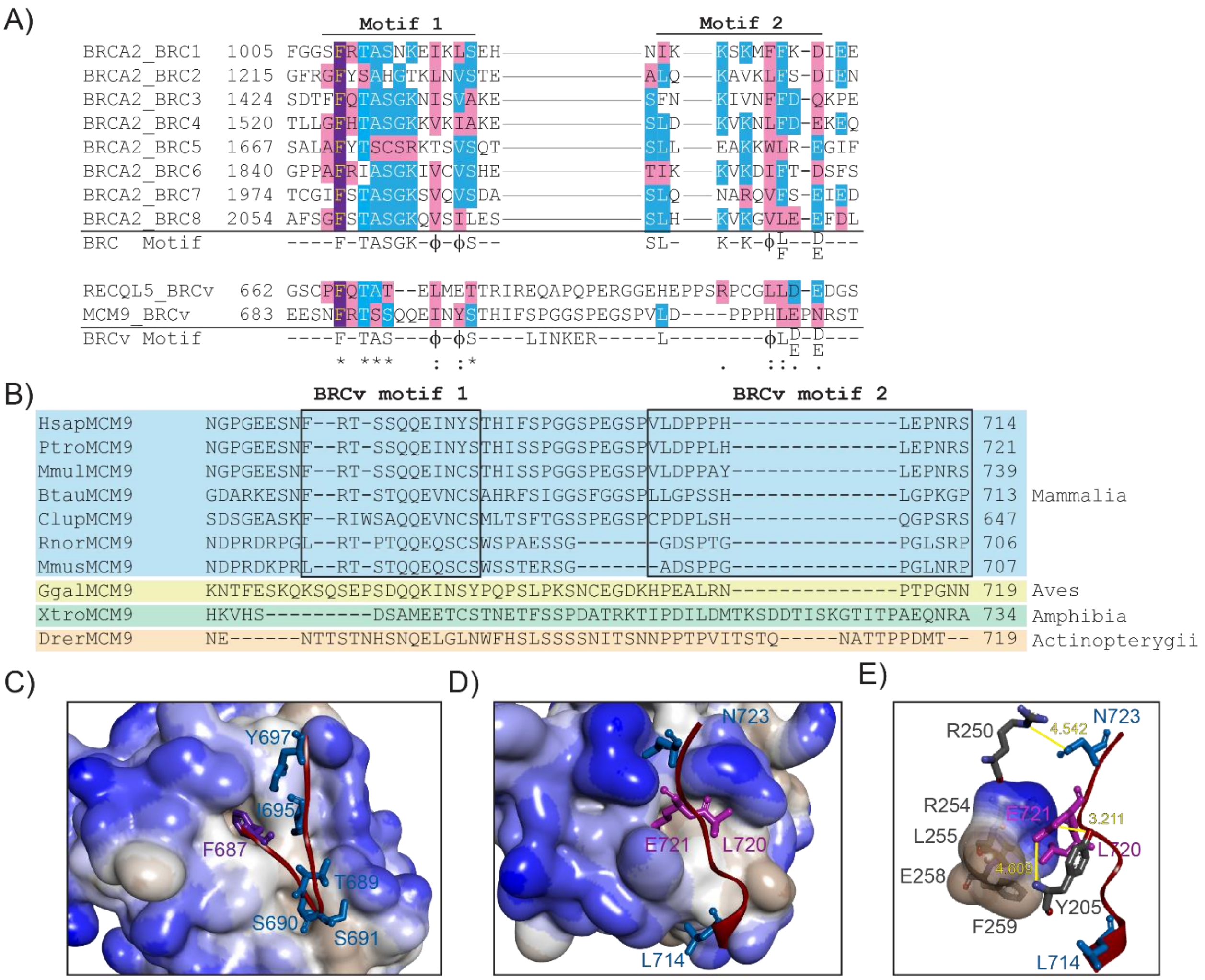

Supplementary Figure S5. BRCv motif alignment, designation, and structure prediction. A) Full alignment of BRC repeats of BRCA2 showing the consensus BRC motif sequence, the BRCv motif of RecQL5 and MCM9, and a consensus BRCv motif sequence. B) BRCv conservation and alignments of MCM9 across *Mammalia*, *Aves*, *Amphibia*, and *Actinoptergii* species. Closeup of C) Motif 1 and D-E) Motif 2 highlighting important interacting residues for MCM9 and RAD51 with a hydrophobic surface (white – hydrophobic and blue- hydrophilic) from a homology model in **Figure 5C**. Predicted H-bonds are indicated (yellow). RAD51 residues are grey.

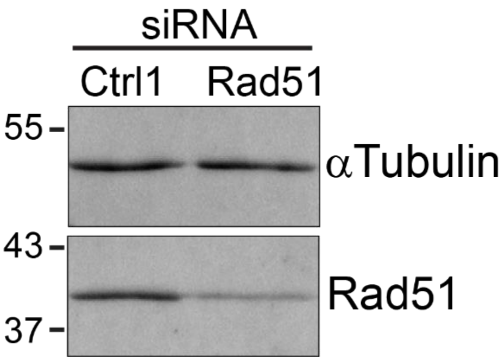

Supplementary Figure S6: siRNA knockdown of Rad51. Western blot of Rad51 in 293T cells with a Ctrl1 or Rad51 siRNA. αTubulin is included as a loading control. Molecular weight markers (kDa) are indicated on the left.

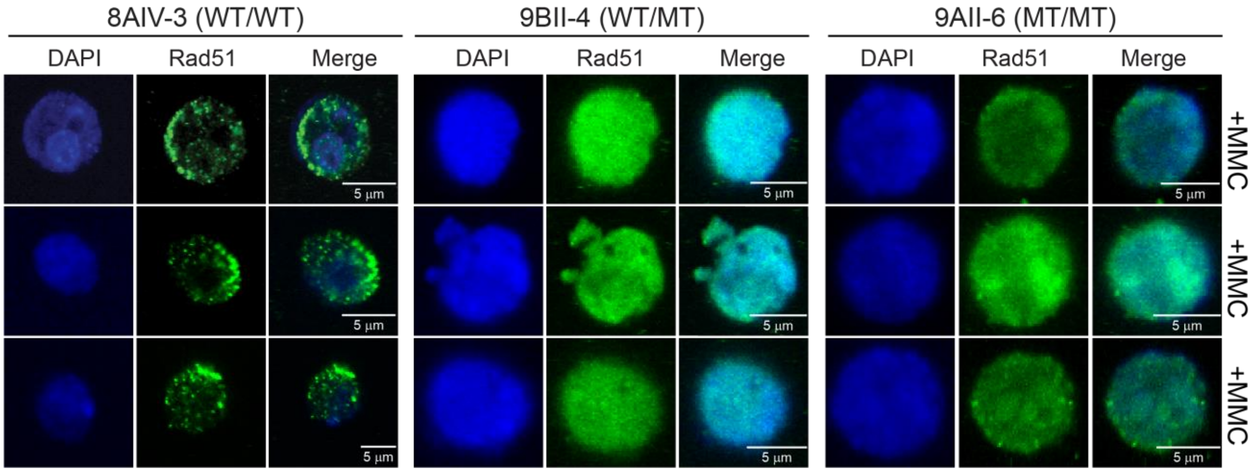

Supplementary Figure S7. Replicates of RAD51 immunofluorescence in MCM9 patient lymphocyte cells treated with MMC. Each panel is a close up of an individual cell. Patient designations as in **Figure 7A-B**.
